# Safety and Efficacy Using the Second-Generation Cryoballoon in Patients With Atrial Fibrillation and a Common Ostium of Inferior Pulmonary Veins

**DOI:** 10.3389/fcvm.2021.683315

**Published:** 2021-09-08

**Authors:** Hai-yang Xie, Xiao-gang Guo, Jian-du Yang, Jia-hui Li, Yan-qiao Chen, Zhong-jing Cao, Qi Sun, Xiao-yao Li, Jian Ma

**Affiliations:** State Key Laboratory of Cardiovascular Disease, Arrhythmia Center, Fuwai Hospital, National Center for Cardiovascular Diseases, Chinese Academy of Medical Sciences and Peking Union Medical College, Beijing, China

**Keywords:** catheter ablation, cryoballoon, atrial fibrillation, pulmonary vein, common ostium

## Abstract

**Background:** Common ostium of inferior pulmonary veins (COIPV) is a kind of pulmonary vein variation. The safety and efficacy of COIPV isolation using the second-generation cryoballoon (CB) ablation remain unknown.

**Methods:** A total of 10 patients with COIPV from a consecutive series of 1,751 patients with atrial fibrillation (AF) were included. Pulmonary vein isolation (PVI) was performed using the second-generation CB.

**Results:** The prevalence of a COIPV was 0.57% in this study. PVI was achieved in all pulmonary veins (PVs) without the need for a touch-up. A segmental freeze strategy was applied for each inferior PV, respectively. The mean number of freeze cycles of inferior PVs was 1.4 ± 0.5 for the left inferior pulmonary vein (LIPV), and 2.0 ± 0.9 for the right inferior pulmonary vein (RIPV). Pulmonary vein potential (PVP) of RIPV could not be monitored in real-time in three cases. Eight of 10 patients (80%) were free from atrial arrhythmias without the use of antiarrhythmic drugs during a follow-up period of 23.6 ± 12.9 months. No procedure-related complications occurred in any of the 10 patients.

**Conclusions:** Common ostium of inferior pulmonary veins is a rare but challenging PV variant. PVI with this unusual anatomic variation using the second-generation 28-mm CB is effective and safe.

## Introduction

Atrial fibrillation (AF), the most common cardiac arrhythmia, affects approximately 1–2% of the entire population (1). Pulmonary vein isolation (PVI) has become a well-accepted strategy and the cornerstone of AF catheter ablation (2, 3). However, the anatomy of the pulmonary vein (PV) varies from person to person. Left common pulmonary vein and right accessory middle vein are the most frequent PV variation (4). The variability of PV anatomy might explain to some extent the presence of PV reconnection after catheter ablation. Although the segmental ablation approach would be adopted to avoid a deep-seating of cryoballoon (CB) in a large common PV ostium, in addition, the total procedure time and the number of applications increased, there was no significant difference in clinical outcomes between variant and non-variant PV anatomy (5–7). A common ostium of inferior pulmonary veins (COIPV), another kind of PV variation, is likely to present in increasing patients with AF. Freedom from atrial tachyarrhythmias would benefit from COIPV isolation (8). However, COIPV isolation performed by radiofrequency catheter ablation was still challenging (8, 9). CB ablation, as an emerging technology in recent years, has shown promising results in achieving freedom from AF as initial therapy in patients with paroxysmal AF (10, 11). Whereas, there was only an isolated case report described previously in the context of PVI using CB ablation (12). Limited data are available about the safety and efficacy of using the second-generation CB ablation in patients with COIPV. Therefore, this study aimed to explore the safety and efficacy of COIPV isolation using the second-generation CB in patients with AF.

## Methods

### Study Population

In a retrospective population of 1,751 consecutive patients with AF who had undergone index ablation by the second-generation CB between May 2016 and October 2019 in Beijing Fuwai Hospital, 10 patients (0.57%) with COIPV determined by multidetector-computed tomography (MDCT) scan were included. Besides, another 40 consecutive patients without COIPV were recruited for comparative analysis between August 2019 and October 2019. All the patients provided written informed consent before the CB ablation. This study was approved by the Institutional Review Board of Fuwai Hospital.

### Preprocedural Management

Antiarrhythmic drugs were discontinued for five half-lives. Transthoracic echocardiography was used to evaluate left atrial (LA) size and left ventricular ejection fraction (LVEF) in all the patients. Transesophageal echocardiography before the procedure was performed to exclude the presence of intracavitary thrombi, and an MDCT scan was performed to analyze the detailed LA and PV anatomy.

### Definitions

The presence of a COIPV was defined as a coalescence of left and right inferior PV (RIPV) before the insertion into the LA (13). The ostium was defined as the point of inflection between the LA wall and the PV wall. The length of the common trunk was defined as a distance from the ostium to the bifurcation.

### CB Ablation of PVs

The procedure was performed as previously described (14). In brief, all cryoablation procedures were performed under deep sedation with continuous infusion of midazolam and fentanyl. After achieving LA access guided by fluoroscopy, single 3-min cryoablation was performed for each vein with the 28-mm CB when optimal PV occlusion was achieved by a “proximal-seal” technique. Isolation of PVs was confirmed by using the inner lumen mapping catheter (Achieve, Medtronic, Minneapolis, MN). Bonus cryoablation was given for the PV when the time to isolation (TTI) was more than 60 s. A segmental freeze strategy would be performed if optimal PV occlusion was difficult to realize. If the balloon nadir temperature exceeded −60°C, the ablation would be terminated. During cryoablation of the right-sided PVs, high-output right phrenic stimulation (1,500 ms; 20 mA) was performed using a quadripolar catheter within the superior vena cava (SVC). When loss of pacing capture occurred, the ablation was immediately terminated.

### Follow-Up

After discharge from the hospital, patients were scheduled for follow-up visits at 1, 3, 6, and 12 months. For each visit, 24-h Holter ECG recordings were performed to evaluate the atrial arrhythmias. First 3 months after the index procedure was considered as the blanking period. Recurrence was defined as any documented atrial episodes with a duration longer than 30 s.

### Statistical Analysis

Continuous variables were described as median or mean ± SD and categorical variables were expressed as percentages. Continuous variables were compared using the Student's *t*-test. A two-tailed value of *P* < 0.05 was considered statistically significant. All the statistical analyses were performed using SPSS 19.0 (IBM Corp., Armonk, NY, USA).

## Results

### Baseline Characteristics

**Figure 1:**
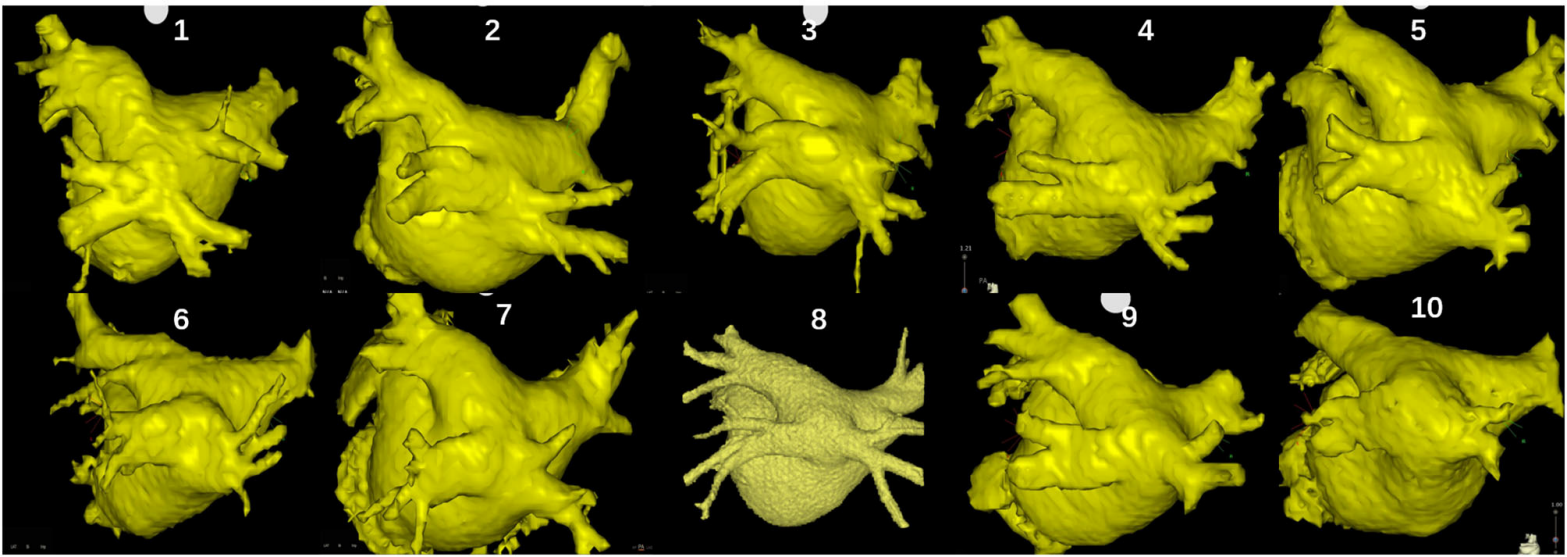
Three-dimensional computer tomography images of the left atrium and pulmonary veins (PVs) of the 10 patients.

A total of 10 (0.57%; nine men; mean age 53 ± 8 years) in 1,751 patients undergoing AF ablation were found to have a COIPV before ablation. The average diameter of the common ostium and LA was 32.8 ± 3.6 mm and 40.8 ± 3.6 mm, and the average length of the common trunk was 9.5 ± 3.4 mm. No structural heart diseases were found in all patients. Characteristics of the patients are described in Table 1. Three-dimensional computer tomography images of LA and PVs gave a better impression of COIPV (Figure 1).

**Table 1:** Baseline characteristics.

**Patients**	***N* = 10**
Paroxysmal atrial fibrillation	9 (90)
Age, y	53 ± 8
Male sex (%)	9 (90)
BMI	27.7 ±2.8
CHA2DS2-VASc (score)	0.5 ± 0.8
Hypertension, n (%)	2 (20)
Diabetes mellitus, n (%)	0 (0)
Previous stroke, n (%)	0 (0)
LA diameter, mm	40.8 ± 3.6
LVEF (%)	62.9 ± 1.9

*Values are expressed as mean ± SD or as n (%).BMI, body mass index; LA, left atrial; LVEF, left ventricular ejection fraction*.

In five of the 10 patients, the esophagus was kept closely in touch with the ostium of the left inferior PV (LIPV). Two cases were exposed to the posterior junction part of the two inferior PVs, and in the remaining three patients, the esophagus contacted the LIPV main branch but stayed away from the ostium.

### Procedural Parameters of PVI

**Figure 2:**
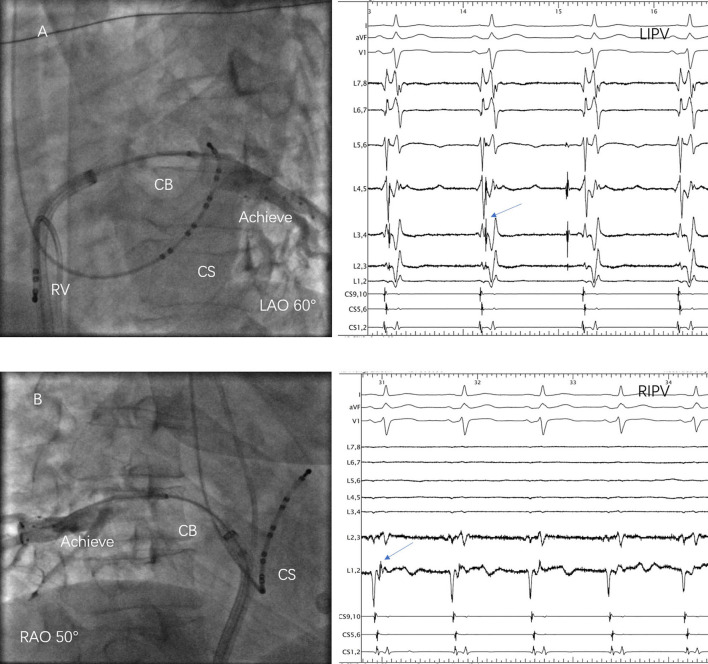
Cryoballoon (CB) ablation of the common ostium of inferior pulmonary veins (COIPV). **(A)** The 28-mm CB was positioned at the ostium of left inferior pulmonary vein (LIPV) and the pulmonary vein (PV) potentials (arrow) were recorded in the Achieve catheter; **(B)** The 28-mm CB was positioned at the ostium of right inferior pulmonary vein (RIPV), LIPV, and the PV potentials (arrow) were recorded in the Achieve catheter.

Although there was a COIPV, the common ostium could not be optimally occluded by the 28-mm CB. Thus, a segmental freeze strategy was adopted (Figure 2). A total of 40 PVs were targeted in the 10 patients. CB ablation started at left superior PV (LSPV), followed by LIPV, right superior PV (RSPV), and RIPV. PVI was achieved without the need for a touch-up in all PVs. Real-time monitor of PVP was difficult to realize in three RIPV, but isolation was confirmed after ablation. A mean number of 8.5 ± 2.6 CB freezes were applied per patient and 2.6 ± 1.1, 1.4 ± 0.5, 2.5 ± 1.6, and 2.0 ± 0.9 for LSPV, LIPV, RSPV, and RIPV, respectively. Procedural and biophysical characteristics are displayed in Table 2.

**Table 2:** Procedural and biophysical characteristics.

Mean total procedural time (minutes)	53.6 ± 7.4
Mean fluoroscopy time (minutes)	13.9 ± 3.3
Mean number of applications, n	8.5 ± 2.6
LSPV	
Mean number of freezes, n	2.6 ± 1.1
Time to isolation (seconds)	39.7 ± 6.2
Temperature at isolation (?C)	−36.7 ± 3.8
Nadir temperature (?C)	−49.4 ± 5.3
LIPV	
Mean number of freezes, n	1.4 ± 0.5
Time to isolation (seconds)	54.0 ± 22.2
Temperature at isolation (^°^C)	−34.3 ± 4.8
Nadir temperature (^°^C)	−39.9 ± 3.3
RSPV	
Mean number of freezes, n	2.5 ± 1.6
Time to isolation (seconds)	65.2 ± 32.3
Temperature at isolation (°C)	−39.7 ± 5.4
Nadir temperature (°C)	−51.5 ± 2.7
RIPV	
Mean number of freezes, n	2.0 ± 0.9
Time to isolation (seconds)	50.7 ± 25.7
Temperature at isolation (°C)	−32.6 ± 11.1
Nadir temperature (°C)	−44.2 ± 7.4

*Values are expressed as mean ± SD. LSPV, left superior pulmonary vein; LIPV, left inferior pulmonary vein; RSPV, right superior pulmonary vein; RIPV, right inferior pulmonary vein*.

Compared to those without COIPV, a higher nadir temperature was observed in patients with COIPV in LIPV (−39.9 ± 3.3°C vs. −44.9 ± 5.5°C; *P* < 0.05), and the same result observed in RIPV (−44.2 ± 7.4°C vs. −50.7 ± 4.2°C; *P* < 0.05). However, there was no significant difference in TTI (−34.3 ± 4.8 s vs. −44.0 ± 22.2 s; *P* = 0.176) for LIPV and (−50.7 ± 25.7s vs. −55.3 ± 32.1 s; *P* = 0.725) for RIPV.

### Follow-Up

No procedure-related complications occurred in any of the 10 patients. Eight of them (80%) were free from atrial arrhythmias without the use of antiarrhythmic drugs during a follow-up period of 23.6 ± 12.9 months. A coronary computed tomography angiography was performed in two patients during the follow-up. No PV stenosis and atrial esophageal fistula (AEF) were detected. In addition, the other patients had no signs or symptoms of AEF, such as fever, chest discomfort, nausea, vomiting, dysphagia, odynophagia, hematemesis, and melena.

## Discussion

### Major Findings

To the best of our knowledge, this is the first study that examines the safety and efficacy of COIPV isolation using second-generation CB in patients with AF. In this study, we found that (1) COIPV was present in 0.57% of the patients included in this study. (2) CB ablation of COIPV was feasible and safe, with acute isolation in 100% of these veins. (3) Manipulation in the set of these patients was not greatly different compared with those without COIPV.

### COIPV Ablation

The two most common PV anomalies are a right accessory middle vein and a left common pulmonary vein, of which optimal PV occlusion was the challenge in a large common ostium. The segmental ablation approach was, thus, adopted to achieve the PV isolation. Whereas, this study demonstrated another variation in the pattern of pulmonary venous drainage. Because of the low prevalence of COIPV, there were only a few case reports of catheter ablation in this subset of patients.

Yu et al. (8) had presented a series of cases who underwent PVI using a “tricircle” catheter ablation strategy, along with the circumferential lines or segmental PV ostium ablation. PVI was acquired in 90% of patients. Furthermore, Squara et al. (2014) (9) demonstrated another strategy isolating the entire posterior wall with a single ring of ablation lesions, which could minimize the lesions on the posterior wall and the risk of esophageal injury. Radiofrequency catheter ablation applied to COIPV had been proven available to achieve PVI. However, during ablation, the catheter tip was difficult to stabilize on the ridge between the two inferior PVs (8). Extensive ablation lesions were required to encircle the PV ostium. Therefore, for one thing, this might pose a challenge to achieve the entire PVI. For another thing, the procedure-related risks and the occurrence of PV reconnection might increase.

The second-generation CB has been widely used to perform PVI due to its efficacy and safety (12, 15). Compared with the first-generation CB, a larger and more uniform zone of freezing was on the surface of the balloon due to the number of injection ports and the maximal cryo-refrigerant flow improvement (16). The second-generation CB ablation could be more consistent in ablation delivery surrounding the PV ostium and less prone to generate conduction gaps in the ablation area (17). As a result, using the second-generation CB tends to receive a better clinical outcome (16), which is especially meaningful to those anatomic variations for avoiding repeat ablation. However, whether CB ablation technology could facilitate COIPV isolation and improve the safety of the procedure remains unknown. Defaye et al. (12) initially reported a case that PVI was achieved by CB ablation without any complications. In this series, it was proved that COIPV could be successfully isolated using second-generation CB without adverse events. PVI was achieved in all the PVs without the need for a touch-up. After a mean follow-up period of about 24 months, 80% of patients were free from AF. Therefore, the electrical isolation of COIPV using the second-generation CB was considered feasible and effective.

### Procedural Behavior of the CB Ablation in the Setting of COIPV

Although COIPV shared a common inferior ostium, the 28-mm CB could not optimally occlude the ostium in these 10 cases, which might be explained by their large ostium and short trunk. The previous study (7) on the left common pulmonary vein had demonstrated that single-shot freeze was associated with a smaller ostial area and a long trunk (>17 mm). Thus, segmental ablation was applied to each inferior PV, respectively. Moreover, RIPV appeared more midline comparing to those without COIPV and fused with LIPV in the proximal portion, but the location of LIPV was not much different. Subsequently, imaging in a larger degree right anterior oblique view was needed when right pulmonary venography and ablation (Figure 2B). During the procedure, once “Achieve” was difficult to access to RIPV, it could be sent into LIPV first and followed by the “FlexCath” sheath. Then rotated the sheath slightly posteriorly, retreated “Achieve” at the same time and then sent it into RIPV. Besides, with such a great degree of angle between RIPV and LA, the “FlexCath” sheath was sometimes difficult to be coaxial with the CB. Therefore, sending “Achieve” deeper into RIPV might avoid it happening. This manipulation was applied to stabilize the CB and optimally occlude the ostium in three cases during ablation. However, PVP could not be monitored in real-time, PVI was confirmed only after ablation application. Over the procedure, manipulation in the set of these patients was not greatly different compared with those without COIPV, including the atrial septal puncture.

### Safety Performance

Anatomically, the LIPV ostium has the closest relationship with the esophagus. Although AEF formation associated with CB ablation is very rare, John et al. (18) reported that eight of 10 AEF were relative to the LIPV. The potential for esophageal damage should be cautious. In this study, the esophagus of half of the patients was kept closely in touch with the LIPV ostium. Two cases were exposed to the junction part of the two inferior PVs, where the frozen area may overlap. The previous study had demonstrated that the longer duration of balloon inflation was significantly associated with AEF (18), and the direct cooling with lower temperature was considered to play a major role in AEF (19). Besides, Fürnkranz et al. (20) demonstrated that the luminal esophageal temperature might continue to decrease for a short period of ablation interruption. In this study, a higher nadir temperature and fewer CB freezes duration were observed, which might avoid AEF happening in all the patients. Besides, repeated ablation at the same site serially was severely restricted during the procedures, especially in those where esophaguses were close to LIPV. Therefore, the “proximal-seal” technique to prevent a precipitous drop in balloon temperatures and the avoidance of continuous ablating at the same PV are significant to protect the esophagus.

### Clinical Implication

The use of second-generation CB is increasing worldwide and the manipulation in patients with COIPV was not greatly different compared with those without COIPV. Pulmonary vein isolation with such unusual variation using second-generation CB might become a promising alternative ablation procedure. A computed tomography scan before the procedure can provide information for planning the ablation strategy and understanding the adjacent anatomy.

### Study Limitations

Given the rarity of COIPV, only 10 cases were included in this retrospective, single-center study. Further studies like a multicenter dataset based on case series are needed to confirm our findings. The endoscope was not used to confirm esophageal injury before and after the procedure.

## Conclusions

The common ostium of inferior pulmonary veins is a rare but challenging PV variant. PVI with this unusual anatomic variation using the second-generation 28-mm CB is effective and safe.

## Data Availability Statement

The raw data supporting the conclusions of this article will be made available by the authors, without undue reservation.

## Ethics Statement

The studies involving human participants were reviewed and approved by Institutional Review Board of Fuwai Hospital. The patients/participants provided their written informed consent to participate in this study.

## Author Contributions

H-yX and X-gG conceived the study. QS and X-yL critically revised the article. Y-qC and Z-jC collected the data. J-dY and J-hL analyzed the data. All authors contributed to the article and approved the submitted version.

## Funding

This study was supported by a grant from the National Natural Science Foundation of China (#81670309).

## Conflict of Interest

The authors declare that the research was conducted in the absence of any commercial or financial relationships that could be construed as a potential conflict of interest.

## Publisher's Note

All claims expressed in this article are solely those of the authors and do not necessarily represent those of their affiliated organizations, or those of the publisher, the editors and the reviewers. Any product that may be evaluated in this article, or claim that may be made by its manufacturer, is not guaranteed or endorsed by the publisher.

## Abbreviations

CB: cryoballoon
CS: coronary sinus
RV: right ventricular
LAO: left anterior oblique
RAO: right anterior oblique
LIPV: left inferior pulmonary vein
RIPV: right inferior pulmonary vein.
